# Aurophilicity as a structure-directing principle in hyperchlorinated Au_n_Cl_n+5_^-^ (2 ≤ n ≤ 7) clusters

**DOI:** 10.3389/fchem.2026.1986508

**Published:** 2026-10-01

**Authors:** Yuqing Chen, Shiyin Xu, Shuo Wang, Shuqi Li, Filip Veljković, Suzana Veličković, Xianglei Kong

**Affiliations:** 1 State Key Laboratory of Elemento-Organic Chemistry, Frontiers Science Center for New Organic Matter, College of Chemistry, Nankai University, Tianjin, China; 2 ‘‘VINCA” Institute of Nuclear Sciences - National Institute of the Republic of Serbia, University of Belgrade, Belgrade, Serbia; 3 Tianjin Key Laboratory of Biosensing and Molecular Recognition, College of Chemistry, Nankai University, Tianjin, China

**Keywords:** aurophilicity, binding energy, gold chloride cluster, mass spectrometry, structure

## Abstract

This study addresses the gap concerning the structural landscape of experimentally observed high-chloride Au_n_Cl_n+5_^-^ cluster anions, whose stable geometries, aurophilic interaction patterns, thermodynamic stability, and growth pathways remain largely unexplored compared to well-characterized low- and medium-chloride analogues. To resolve this, we systematically investigate Au_n_Cl_n+5_^-^ (n = 2–7) using laser desorption/ionization mass spectrometry (LDI-MS) and density functional theory at the TPSSh/aug-cc-pVTZ/ECP60MDF level. The results show that elevated laser intensity preferentially stabilizes these chlorine-rich clusters. Except for the smallest cluster (Au_2_Cl_7_^−^), the global minima adopt nearly planar zigzag geometries, where terminal gold atoms are in a tetracoordinate Au(III) state and internal gold atoms are in an Au(I) state. Bond-length analysis reveals that only non-terminal Au–Au distances are significantly shorter than the van der Waals sum, indicating aurophilic attraction, while terminal distances show no such interaction. Aurophilicity emerges at Au_4_Cl_9_^−^ and rapidly stabilizes for larger clusters. Natural bond orbital (NBO) and electron localization function (ELF) analyses attribute this interaction to 6s/5d orbital delocalization between adjacent Au(I) centers, with no contribution from Au(III)–Au(I) pairs. Comparison with lower-chloride series (Au_n_Cl_n+1_^−^ and Au_n_Cl_n+3_^−^) indicates that the core thermodynamic trend remains unchanged, although higher laser fluence favors chlorine-rich species via thermodynamically favored addition of two Cl atoms. This study highlights the structure-directing role of aurophilicity in hyperchlorinated gold clusters and provides a comprehensive understanding of their size-dependent structural evolution.

## 1 Introduction

Gold and its compounds play pivotal roles in catalysis, supramolecular chemistry, materials science, and optoelectronic devices (Hashmi, 2007; Zhang et al., 2012; Adak, 2025; Mishra and Patil, 2023; Wang et al., 2021; Izadyar et al., 2018). The unique physicochemical properties of gold stem largely from pronounced relativistic effects (Bartlett, 1998; Theilacker et al., 2015), which profoundly modulate its electronic structure and distinguish it from its lighter congeners (Pyykkö, 2005; Pyykkö, 2008). Recently, nanogold catalysis has emerged as a vibrant research Frontier (Pyykkö, 2004). Within this context, gold clusters serve as crucial intermediates bridging molecular complexes and nanoparticles, offering invaluable insights into the nature of active sites and underlying reaction mechanisms.

Among the panoply of weak interactions in gold chemistry, the aurophilic interaction—a specific attraction between closed-shell Au(I) centers—has garnered exceptional interest. The phenomenon was first identified in 1988 by Schmidbaur and co-workers through the observation of anomalously short Au–Au distances in gold(I) compounds (Dipl et al., 1988), subsequently leading to the coining of the term “aurophilicity” (Schmidbaur and Schier, 2012). Pyykkö and colleagues later provided a seminal theoretical framework, attributing this interaction to a relativistic-enhanced dispersion force with an energy magnitude intermediate between that of van der Waals forces and covalent bonds (Pyykkö, 2005; Pyykkö, 2008; Pyykkö, 2004). Subsequent reviews by Sculfort and Braunstein, (2011) and by Schmidbaur and Schier, (2012) have further underscored the pivotal structure-directing role of aurophilicity in constructing complex heterometallic and supramolecular architectures, cementing its status as a cornerstone concept in modern gold chemistry.

Gold halides serve as pivotal precursors and reactive intermediates in gold catalysis, and their structural characterization offers vital insights into the fundamental chemistry of gold (Lemke, 2014; Baker et al., 2008; Davies et al., 2016). Schwerdtfeger and co-workers conducted a comparative study on the structures and stabilities of group-11 tetramers (M_4_X_4_; M = Cu, Ag, Au; X = F–I), revealing that relativistic effects and aurophilic attractions endow gold halides with distinct structural motifs (Schwerdtfeger et al., 2004). Extending this work, Rabilloud examined the geometric and electronic structures of coinage-metal halide clusters (M_n_X_n_; M = Cu, Ag, Au; X = Br, I; n = 1–6), further highlighting the decisive role of aurophilicity in dictating cluster geometry (Rabilloud, 2012). Using Fourier-transform ion cyclotron resonance mass spectrometry (FT-ICR MS) coupled with MP2 theory, Lemke et al. elucidated the valence distribution of Au(III) and Au(I) within gaseous gold chloride clusters, uncovering a rich and complex gas-phase chemistry (Lemke, 2014). From a mechanistic perspective, Barngrover and Aikens explored the theoretical conversion pathway from Au(III) chlorides to Au(I) thiolates, shedding light on the nucleation mechanisms of gold nanoparticles (Barngrover and Aikens, 2012). Beyond simple halides, the structural landscape extends to exotic superhalogen species. For instance, Li et al. reported the synthesis and characterization of FLi_4_X_4_^-^ (X = Cl, Br, I) anions featuring a rare planar tetracoordinate fluorine center (Li et al., 2024). Recent advances in superhalogen chemistry continue to expand the scope of halide-containing cluster systems, underscoring their structural versatility (Srivastava, 2023). Complementarily, experimental and theoretical evidence has revealed that gold can exhibit intriguing halogen-like chemical behaviors in certain bimetallic cluster environments (Long et al., 2024).

Gas-phase cluster studies have further illuminated the nuanced interplay between bonding and aurophilicity. Using photoelectron spectroscopy complemented by theoretical calculations, Li et al. identified bond-bending isomerism in Au_2_I_3_^−^, highlighting a delicate competition between covalent bonding and aurophilic attraction (Li et al., 2016). Wang subsequently reviewed covalent gold chemistry, attributing both Au–Cl covalency and aurophilic interactions to relativistic-induced orbital hybridization (Wang, 2010). Focusing on anionic chlorides, Ma et al. systematically characterized Au_n_Cl_n+1_^-^ (n = 1–7) via FT-ICR MS and MP2 calculations (Ma et al., 2019a). Their work established that the global minima adopt characteristic zigzag motifs and revealed a distinct size effect: Au–Au distances progressively contract and converge with increasing nuclearity. Crucially, they demonstrated that relativistic effects are indispensable, as their omission in non-relativistic calculations resulted in significantly elongated Au–Au contacts (Ma et al., 2019a). In subsequent studies, Ma et al. reported enhanced electron affinities in Au_2n_Cl_n_ clusters upon chlorine doping (Ma et al., 2019b), while Xu et al. extended the structural landscape to Au_n_Cl_n+3_^−^ (n ≤ 7). The latter work identified similar zigzag frameworks but noted that the inclusion of an Au(III) site critically modulates cluster geometry and growth pathways (Xu et al., 2024). Complementing these theoretical insights, Veljković et al. experimentally assessed the stability of Au_n_Cl_n+m_^−^ (n = 1–5; m = 1, 3, 5, 7) using laser desorption/ionization (LDI) mass spectrometry, observing marked variations in abundance distributions contingent on chloride stoichiometry (Veljković et al., 2025).

While the structure–property correlations of low- and medium-chloride gold clusters are well-established, the structural landscape of the experimentally observed high-chloride Au_n_Cl_n+5_^-^ series remains largely uncharted. Critical questions regarding their stable geometries, aurophilic interaction patterns, thermodynamic stability, and growth pathways persist. Herein, we bridge this knowledge gap by systematically investigating Au_n_Cl_n+5_^-^ cluster anions, integrating laser desorption/ionization mass spectrometry (LDI-MS) with density functional theory (DFT) calculations. Our combined approach enables the determination of global minima, a detailed analysis of the size-dependent evolution and progresses of aurophilic interactions. By comparing with the lower-chloride Au_n_Cl_n+1_^−^ and Au_n_Cl_n+3_^−^ series, we elucidate how increasing chloride content and the resulting coordination environment govern the geometric and electronic structure of these clusters.

## 2 Experimental

The experiments in this work were performed with a MALDI-TOF mass spectrometer (Voyager-DE PRO Sciex, Foster City, CA, United States). An ultraviolet N_2_ laser (wavelength 337 nm, pulse width 3 ns, repetition rate 20.00 Hz) was used to obtain clusters. The number of laser shots was 300 per mass spectrum. The instrumental parameters were as follows: acceleration voltage 20,000 V, grating voltage 94%, and delayed extraction time 100 ns.

Mass spectra were acquired in the absence of a conventional organic matrix or any other matrix, using the LDI technique. The sample consisted of an aqueous HAuCl_4_ solution at a concentration of 2.5 g dm^-3^ (99.99%, CAS No. 12453-07-1), prepared in deionized water (Millipore) immediately prior to the measurements. An aliquot of 0.5 μL of the HAuCl_4_ solution was deposited onto a spot of a stainless-steel target plate and subsequently analyzed by LDI-TOF mass spectrometry.

### 2.1 Computation

Global minimum searches for Au_n_Cl_n+5_^-^ (n = 2–7) clusters were conducted using the NKCS program (Zhou et al., 2021), supplemented by manually constructed isomers exhibiting structural homology along possible growth pathways. Geometry optimizations were subsequently performed using Gaussian 09 at the TPSSh level, employing the pseudopotentials ECP60MDF relativistic pseudopotential and its associated MDF60 basis set for Au, alongside the aug-cc-pVTZ basis set for Cl (Figgen et al., 2005; Peterson and Puzzarini, 2005; Schwerdtfeger et al., 1998; Woon and Dunning, 1993). Frequency analyses confirmed all optimized structures as true minima on their respective potential energy surfaces (PESs). Finally, bonding characteristics were probed via ELF (Electron Localization Function) (Lu and Chen, 2011), all executed within the Multiwfn package (Lu and Chen, 2012). ZPE (Zero-point energy) corrections derived from these frequency analyses were included for all reported binding energies. All binding-energy values provided in this work correspond to zero-point-corrected energies. No Boys-Bernardi counterpoise correction for basis-set superposition error (BSSE) was performed in the present work. Although the large augmented triple-zeta basis set aug-cc-pVTZ can substantially mitigate BSSE effects, neglecting BSSE generally slightly overestimates the magnitude of calculated binding energies. Full counterpoise-corrected energy calculations are left for future higher-level theoretical investigations.

## 3 Results and discussion

Our previous studies demonstrated that Au_n_Cl_n+1_^−^, Au_n_Cl_n+3_^−^, and Au_n_Cl_n+5_^−^ clusters (n = 2–5) were identified in the LDI mass spectra over a laser intensity range of 1,200–2,600 a.u. (Veljković et al., 2025). Among the investigated conditions, the optimal laser intensity for the formation and detection of Au_n_Cl_n+5_^−^ clusters was found to be 2,300 a.u. The corresponding mass spectrum is presented in Figure 1.

**FIGURE 1:**
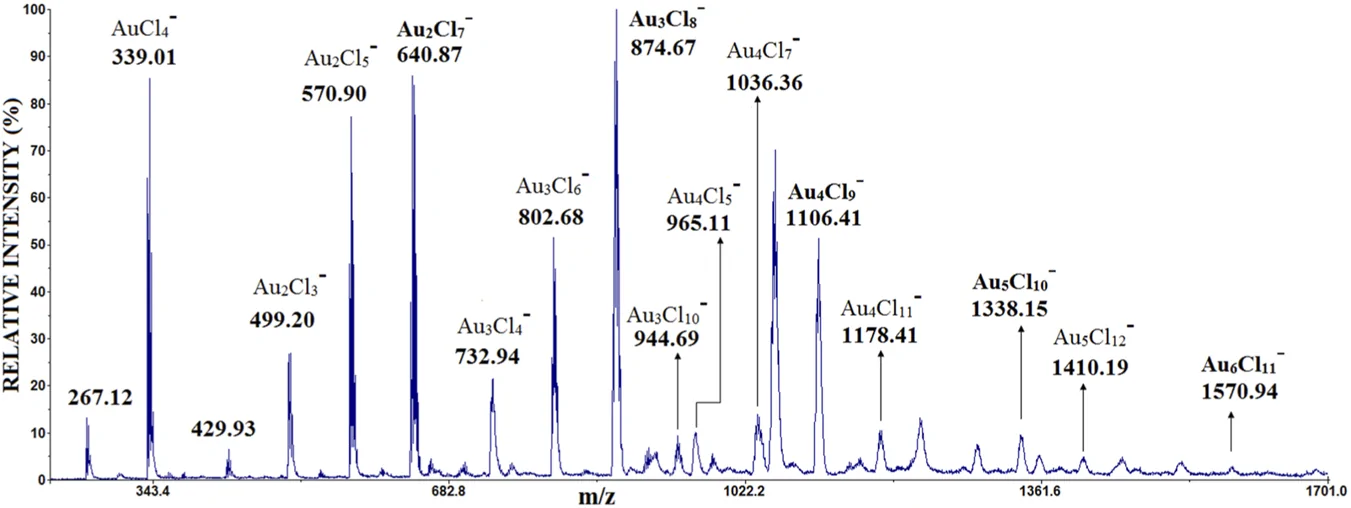
The anions of Au_n_Cl_n+1_^−^, Au_n_Cl_n+3_^−^, and Au_n_Cl_n+5_^−^ clusters obtained in the LDI mass spectrum of HAuCl_4_.

All detected anions are labeled in the spectrum, with the anions belonging to the Au_n_Cl_n+5_^−^ cluster series highlighted in bold (Figure 1). Among the detected species, the Au_3_Cl_8_^−^ anion, belonging to the Au_n_Cl_n+5_^−^ cluster series, exhibits the highest peak intensity. The Au_2_Cl_7_^−^ and Au_4_Cl_9_^−^ anions from the same cluster series also show significant intensities, whereas the Au_5_Cl_10_^−^ and Au_6_Cl_11_^−^ anions are detected with considerably lower intensities (Figure 1).

On the other hand, the relative intensities of the Au_2_Cl_3-7_^−^, Au_3_Cl_4-8_^−^, and Au_4_Cl_5-9_^−^ series obtained under two laser intensities were compared in Figure 2. As shown in Figure 2A, the relative peak intensities follow the order Au_2_Cl_3_^−^ > Au_2_Cl_5_^−^ > Au_2_Cl_7_^−^ at a low laser intensity (1,300 a.u.), whereas this order is reversed at a high laser intensity (2,300 a.u.), with Au_2_Cl_3_^−^ < Au_2_Cl_5_^−^ < Au_2_Cl_7_^−^. For the n = 3 series (Figure 2B), the results differ slightly; the relative peak intensities follow the order Au_3_Cl_4_^−^ > Au_3_Cl_6_^−^ < Au_3_Cl_8_^−^ under low laser intensity, indicating that Au_3_Cl_6_^−^ exhibits a lower relative intensity than both Au_3_Cl_4_^−^ and Au_3_Cl_8_^−^. However, under high laser intensity, the intensity hierarchy shifts to Au_3_Cl_4_^−^ < Au_3_Cl_6_^−^ < Au_3_Cl_8_^−^, with Au_3_Cl_8_^−^ becoming the dominant species. For the Au_4_Cl_5_,_7_,_9_^−^ series, the trends closely mirror those of the Au_3_Cl_4_,_6_,_8_^−^ series (Figure 2C). Taken together, these results suggest that increasing the laser intensity favors the formation of chlorine-rich clusters, particularly those belonging to the Au_n_Cl_n+5_^−^ series.

**FIGURE 2:**
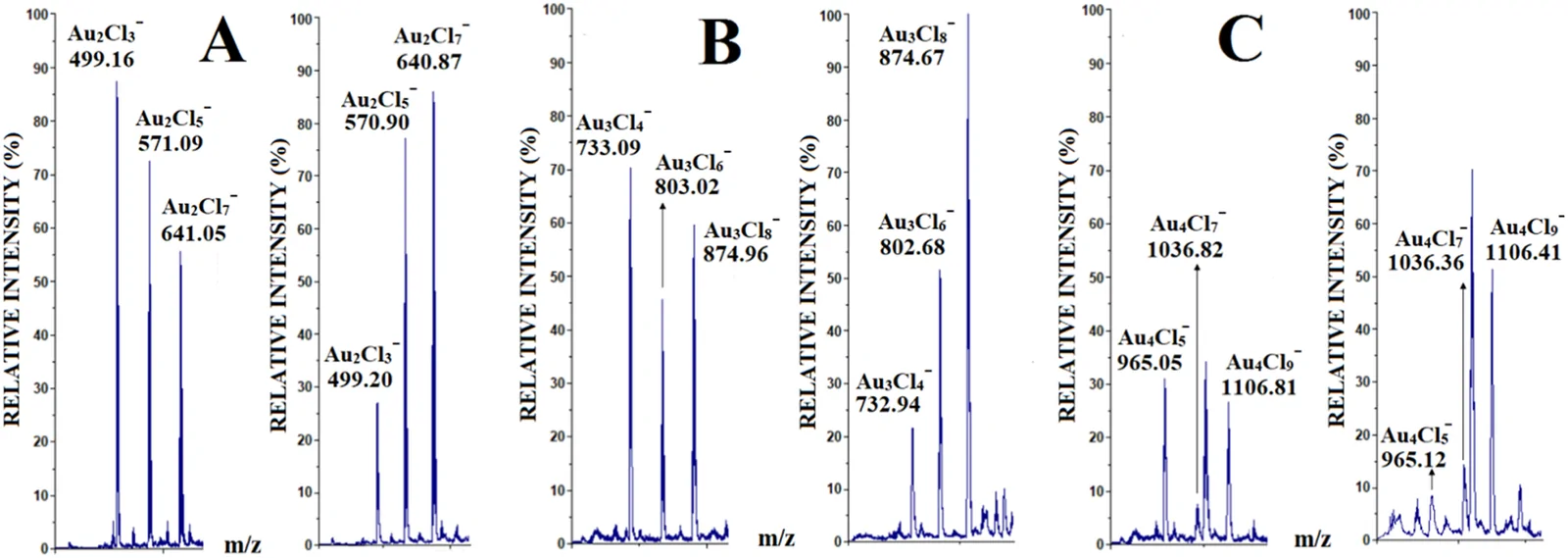
The relative intensities of the Au_n_Cl_n+1_^-^, Au_n_Cl_n+3_^-^, and Au_n_Cl_n+5_^-^ (n = 2, 3, 4) cluster at laser intensities of 1,300 a.u. (left) and 2,300 a.u. (right): **(A)** Au_2_Cl_3_^−^, Au_2_Cl_5_^−^, Au_2_Cl_7_^−^; **(B)** Au_3_Cl_4_^−^, Au_3_Cl_6_^−^, Au_3_Cl_8_^−^; **(C)** Au_4_Cl_5_^−^, Au_4_Cl_7_^−^, Au_4_Cl_9_^−^.

To identify the global minima of Au_n_Cl _n+5_^−^ (n = 2–4) cluster anions, an extensive structural search was conducted, followed by geometry optimizations and energetic comparisons. Figure 3 displays the six most stable isomers identified for these anions.

**FIGURE 3:**
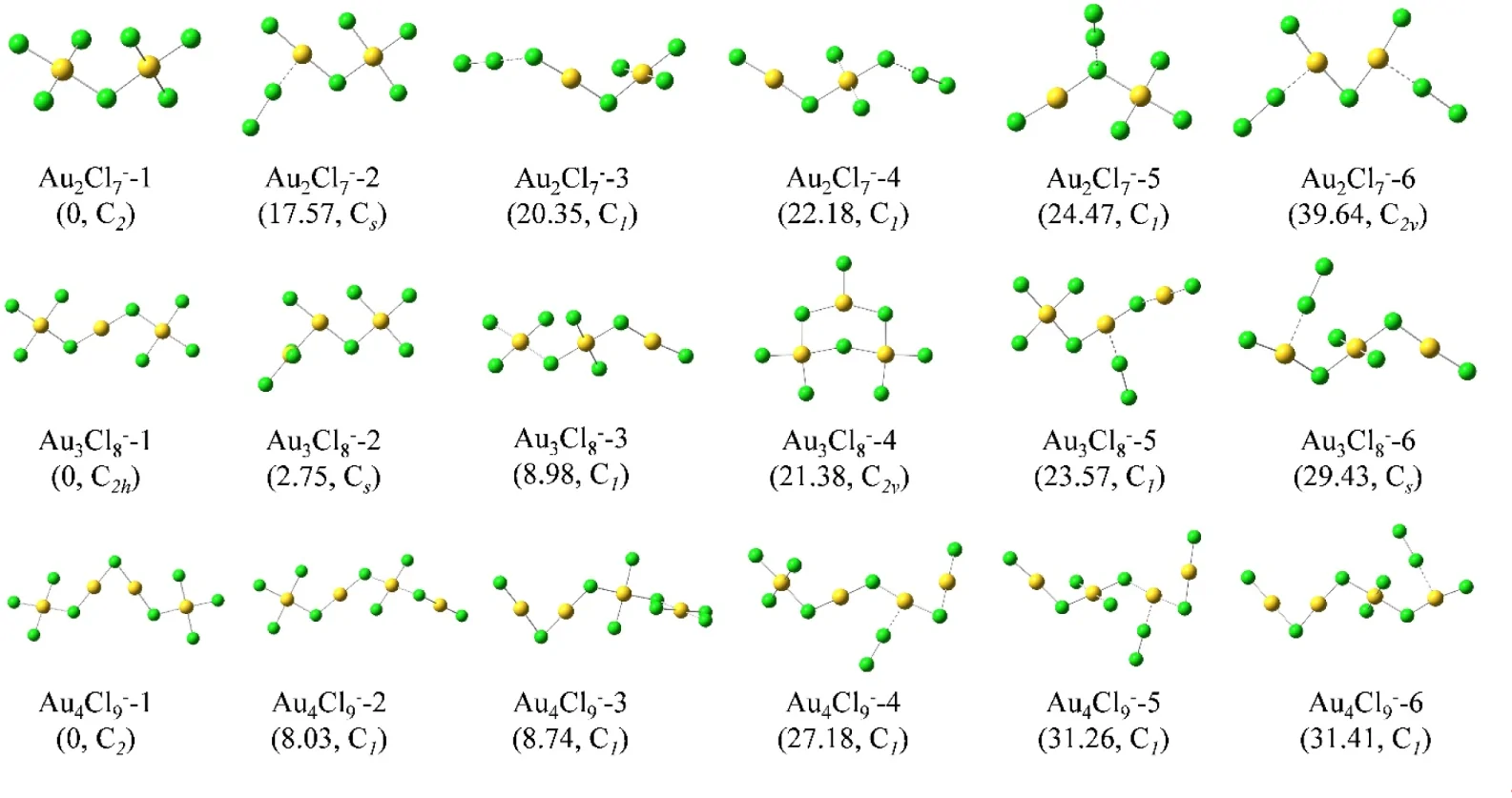
Structures of the top 6 isomers of Au_n_Cl_n+5_^-^ (n = 2–4). Their relative energies (ΔE, in kcal mol^-1^) and symmetry are shown in parentheses.

Most interestingly, the results reveal that the global minima of the series adopt zigzag configurations once again, closely resembling those reported previously for Au_n_Cl_n+1_^−^ and Au_n_Cl_n+3_^−^ (n = 2–4) (Ma et al., 2019a; Xu et al., 2024). Although previous studies reported low-lying isomers in Au_n_Cl_n+1_^−^ and Au_n_Cl_n+3_^−^ containing Au–Au bonds, such motifs are absent in the global minima of Au_n_Cl_n+5_^−^ presented in Figure 3. This absence is likely attributed to the pronounced excess of Cl over Au atoms within these anionic clusters. The most stable isomers are still based on the zigzag skeleton of Au-Cl, with the other Cl atoms adding at opprosite positions vertical to the main chain, while some isomers also append the unit of Cl_2_ to the main chain. And the latter usually has an energy about 20 kcal/mol or more than those of the most stable isomers.

The structural similarity between Au_n_Cl_n+5_^−^ (n = 2–4) and Au_n_Cl_n+1/n+3_^−^ (n = 2–4) implies plausible growth pathways wherein the former evolve from the latter. Concurrently, the structural consistency among Au_n_Cl_n+5_^−^ (n = 2–4) underscores the inherent structural inheritance of these cluster anions. Guided by these insights, we proposed, optimized, and identified candidate structures for the larger analogues Au_n_Cl_n+5_^-^ (n = 5–7), which are characterized by analogous zigzag skeletons and terminal AuCl_4_^−^ units. These geometries are compared in Figure 4. Notably, the most stable isomers across this series share a common architectural motif: the zigzag backbone is invariably flanked by AuCl_4_^−^ units at both termini. This observation highlights the critical role of aurophilic interactions in stabilizing these frameworks. For the less stable isomers of Au_n_Cl_n+5_^−^ (n = 4–7), relocating a single tetracoordinate Au(III) center from a terminal to an internal position incurs an energetic penalty of 6–11 kcal/mol.

**FIGURE 4:**
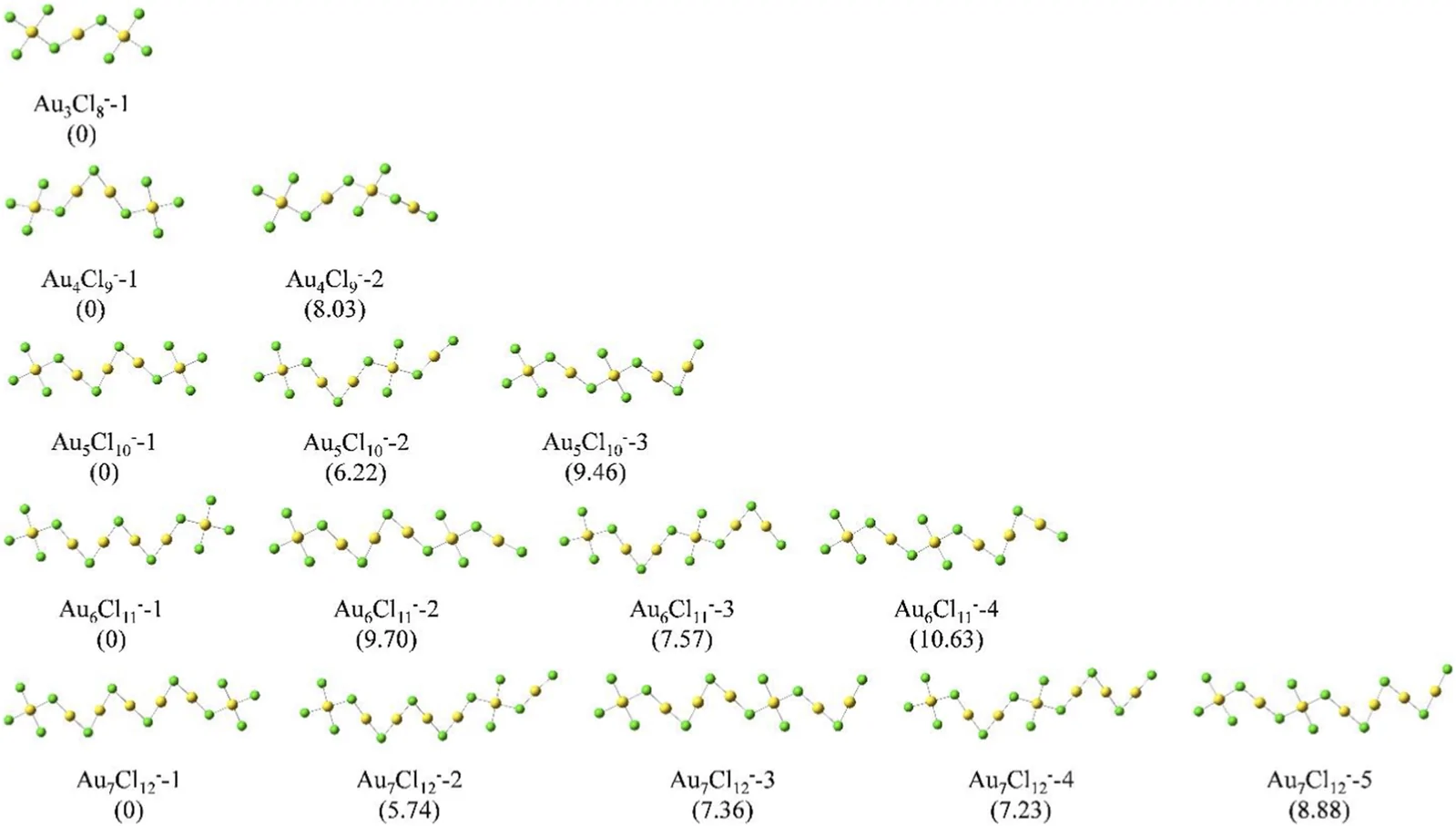
Optimized Zigzag-type geometries of Au_n_Cl_n+5_^-^ (n = 3–7) cluster anions.

Aurophilic interactions within these isomers can be probed by analyzing Au–Au distances. For clarity, we define the “terminal Au–Au distance” as the separation between a terminal Au(III) center and its adjacent Au(I) or Au(III) neighbor, whereas the “non-terminal Au–Au distance” refers to the spacing between two adjacent internal Au(I) centers. Given the sum of the van der Waals radii for gold (3.32 Å; Sculfort and Braunstein, 2011) and the typical covalent Au–Au bond length (2.72 Å; Cordero et al., 2008), characteristic aurophilic interactions are expected to fall within the range of 2.72–3.32 Å.

As listed in Table 1, which summarizes the most stable isomers of Au_n_Cl_n+5_^−^ (n = 2–7), all terminal Au–Au distances (3.82–3.90 Å) exceed the 3.32 Å threshold. This indicates significant steric repulsion among the clustered chlorine ligands, precluding aurophilic bonding at the termini. In contrast, all non-terminal distances reside within a narrow range of 2.90–2.92 Å, confirming robust aurophilic attraction. Notably, these internal distances remain virtually invariant with increasing chain length. Furthermore, the observed non-terminal distances are marginally shorter than those reported for Au_n_Cl_n+1_^−^ and Au_n_Cl_n+3_^−^. This intriguing result suggests that the incorporation of additional chlorine atoms, in fact, serves to slightly enhance the aurophilic attraction between the internal Au(I) centers along the zigzag backbone.

**TABLE 1:** Terminal (average) and non-terminal nearby Au-Au distances in Au_n_Cl_n+5_^-^ (n = 2–7) (in Å).

Structure	Au_2_Cl_7_-1	Au_3_Cl_8_-1	Au_4_Cl_9_-1	Au_5_Cl_10_-1	Au_6_Cl_11_-1	Au_7_Cl_12_-1
Terminal Au-Au	3.89	3.90	3.86	3.85	3.83	3.82
Non-terminal Au-Au	​	​	2.92	2.90	2.90	2.90
	​	​	​	2.90	2.91	2.91
	​	​	​	​	2.90	2.91
	​	​	​	​	​	2.90

Complementary Electron Localization Function (ELF) maps (Figure 5) corroborate these findings. Between terminal Au(III) and adjacent Au(I)/Au(III) atoms, the electron-pair density is virtually nil, reaffirming the lack of aurophilic contact in agreement with the bond-length criteria. Conversely, a distinct localization domain emerges between internal Au(I) pairs. Notably, the area and intensity of this domain increase progressively with cluster size, eventually reaching a plateau for n ≥ 4. This trend provides compelling evidence for the size-dependent saturation of aurophilic interactions within the zigzag backbone.

**FIGURE 5:**
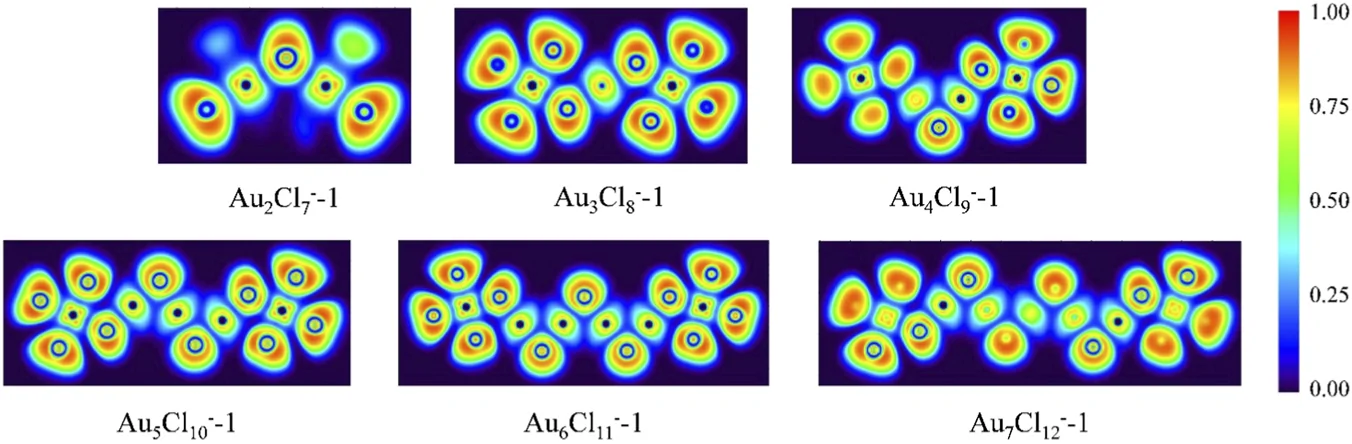
ELF maps of the most stable isomers of Au_n_Cl_n+5_^-^ (n = 2–7).

To gain deeper insight into the nature of aurophilic attraction, Natural Bond Orbital (NBO) analyses were performed on the most stable isomers of Au_n_Cl _n+5_^-^ (n = 2–7), with the results summarized in Supplementary Table S1. The calculations reveal that the two terminal Au(III) centers bear identical positive charges, which are substantially higher than those of the internal Au(I) centers (ca. +0.47 vs. +0.34|e|). Examination of the electron configurations indicates that the higher coordination number and oxidation state of Au(III) result in a more pronounced depletion of 5d electrons, a reduction in 6s occupancy, and increased 6p participation. Crucially, significant 5d/6s orbital delocalization is observed exclusively between adjacent internal Au(I) centers, identifying the electronic origin of the aurophilic interaction. In stark contrast, negligible orbital mixing occurs between terminal Au(III) and neighboring Au(I) centers, consistent with the absence of aurophilic bonding at the termini. (Mendizabal and Pyykkö, 2004). Although NBO analysis provides useful orbital information for the present gold-chlorine clusters, further comprehensive Adaptive Natural Density Partitioning (AdNDP) calculations (Zubarev and Boldyrev, 2008) would be highly desirable to achieve deeper understanding of the multicenter bonding features within these hyperchlorinated gold cluster anions. Beyond aurophilic attraction, diverse weak non-covalent forces can serve as powerful structure-directing factors within heavily-halogenated inorganic systems. Halogen bonding, for instance, may override steric constraints and define final coordination topologies under extreme halogen-rich conditions, as demonstrated by DFT-based topological analyses of halogen-containing coordination assemblies (Yardan et al., 2026). In our hyperchlorinated gold-cluster anions, aurophilicity arising from d10-d10 orbital delocalization dominates the one-dimensional zig-zag backbone assembly. This distinction underscores the tunable competition among multiple weak intermolecular forces that govern geometry in halogen-rich metal-cluster systems.

The binding energy for this series is defined as the energy change for the incremental growth reaction: Au_n-1_Cl_n+4_^-^ + AuCl → Au_n_Cl_n+5_^-^. All computed binding energies are negative, ranging from −188 to −176 kJ/mol, confirming that cluster growth is exothermic and that these anions are thermodynamically viable (Figure 6). With increasing nuclearity, the absolute binding energy exhibits a monotonic decrease before converging asymptotically to a value of approximately −176 kJ/mol. This thermodynamic convergence parallels the trends observed in Au–Au bond lengths and Electron Localization Function (ELF) analyses, suggesting a plateau of aurophilic interactions. Comparative analysis with the previously reported Au_n_Cl_n+1_^−^ and Au_n_Cl_n+3_^−^ series reveals that their thermodynamic behavior—namely, the gradual convergence of binding energy and aurophilic interaction strength—is largely invariant with respect to chloride content.

**FIGURE 6:**
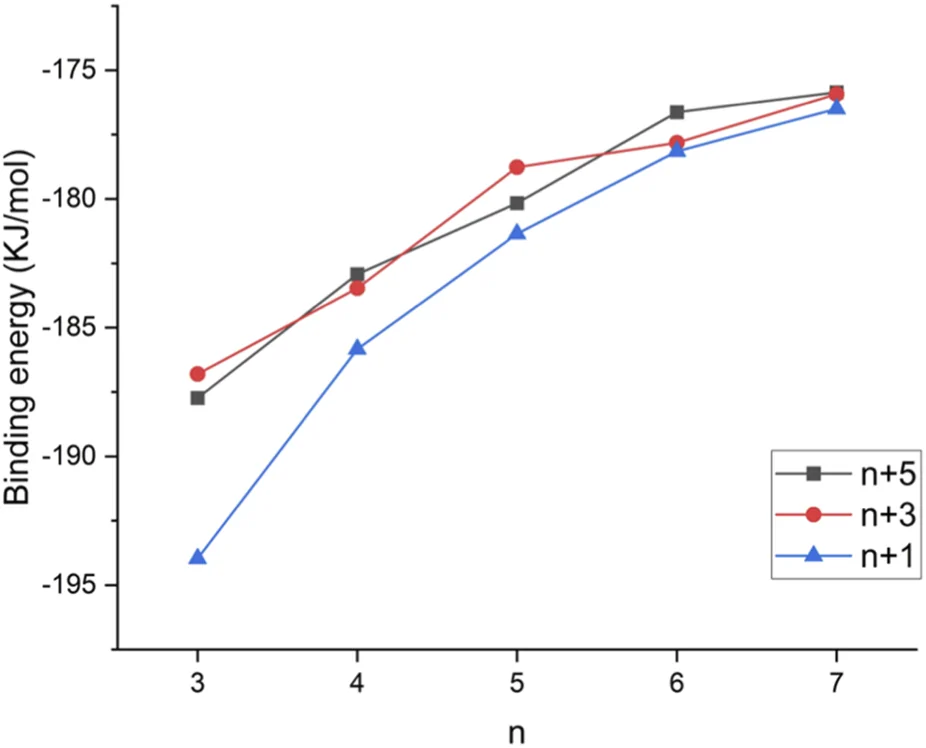
Binding energies of Au_n_Cl_n+1_^-^, Au_n_Cl_n+3_^-^ and Au_n_Cl_n+5_^-^ (n = 3–7).

These results, together with our previous studies, provide a consistent explanation for the observed Au_n_Cl_n+1,n+3,n+5_^−^ series. However, the binding energies displayed in Figure 6 fail to account for the intensity distributions of Au_3_Cl_6_^−^ and Au_4_Cl_7_^−^ shown in Figures 2B,C. Why do these two ions exhibit relatively lower intensities compared to their neighbors at low laser intensity? The underlying reasons are likely complex; nevertheless, clues may be gleaned by examining the most probable formation pathways under the experimental conditions. Taking Au_3_Cl_6_^−^ as an example, it can be formed via the following reactions of Equations 1, 2:

$$Au_{2}Cl_{5}^{-}+AuCl\;\to \;Au_{3}Cl_{6}^{-}$$ (1)

$$Au_{3}Cl_{4}^{-}+2Cl\;\to \;Au_{3}Cl_{6}^{-}$$ (2)

It can also be depleted via further reaction pathways, including Equations 3, 4:

$$Au_{3}Cl_{6}^{-}+AuCl\;\to \;Au_{4}Cl_{7}^{-}$$ (3)

$$Au_{3}Cl_{6}^{-}+2Cl\;\to \;Au_{3}Cl_{8}^{-}$$ (4)

The total intensity of Au_3_Cl_6_^−^ reflects the balance among these reactions. The energies released from the four pathways are −187, −362, −183, and −357 kJ/mol, respectively.

Similarly, Au_3_Cl_8_^−^ can be formed and consumed through the following reactions (Equations 5–8):

$$Au_{2}Cl_{7}^{-}+AuCl\;\to \;Au_{3}Cl_{8}^{-}$$ (5)

$$Au_{3}Cl_{6}^{-}+2Cl\;\to \;Au_{3}Cl_{8}^{-}$$ (6)

$$Au_{3}Cl_{8}^{-}+AuCl\;\to \;Au_{4}Cl_{9}^{-}$$ (7)

$$Au_{3}Cl_{8}^{-}+2Cl\;\to \;Au_{3}Cl_{10}^{-}$$ (8)

Among them, reaction (6) is identical to Reaction (4), while Reactions (5) and (7) are analogous to Reactions (1) and (3). The energies released from Reactions (5) and (7) are −188 and −183 kJ/mol, respectively. In contrast, Reaction (8) contributes very differently compared to Reaction (4). The addition of excess Cl atoms introduces significant steric repulsion within the cluster, rendering Reaction (8) far less thermodynamically favored than Reaction (4). This is verified by the weak signal of Au_3_Cl_10_^−^ in the experimental mass spectra (Figure 1). Consequently, this accounts for the relatively lower peak intensity of Au_3_Cl_6_^−^ observed under low laser intensity. As the laser intensity increases, the Cl atom concentration rises rapidly, causing Reactions (2) and (4) to dominate the process. This results in a marked decrease in the relative intensity of Au_3_Cl_4_^−^ and a corresponding increase in that of Au_3_Cl_8_^−^ under high laser intensity. We note that the above interpretation is built purely upon thermodynamic reaction-energy analysis. Given that these structures exhibit strong inheritance and the reactions do not involve the breaking of chemical bonds—making them very close to barrierless processes—the assumption of thermodynamic control, rather than kinetic control, is reasonable.

The mechanisms outlined above are also applicable to Au_4_Cl_7_^−^. However, Au_2_Cl_5_^−^ exhibits different behavior under low laser intensity, even though a similar reaction scenario exists for Au_2_Cl_7_^−^. This apparent inconsistency may be explained by the formation pathway AuCl_4_^−^ + AuCl → Au_2_Cl_5_^−^ and the high concentration of the AuCl_4_^−^ precursor generated in the laser ionization processes (which can be reflected in Figure 1).

It is reasonable to expect that Au_n_Cl_n+m_^−^ (m > 5) clusters, characterized by an even higher Cl:Au ratio, could also exist, retaining similar yet extended zigzag backbones. Given the potential to modulate the chlorine species concentration during synthesis, the structural flexibility and compositional diversity of these one-dimensional gold–halogen chains may open new avenues for their catalytic activity—or that of related species—in relevant reactions.

## 4 Conclusion

In summary, our results demonstrate that elevated laser intensity preferentially stabilizes clusters of the type Au_n_Cl_n+5_^-^ (n = 2–6) during the laser desorption/ionization of HAuCl_4_. For understanding the structures of these cluster anions observed in, the most stable isomers of Au_n_Cl_n+5_^-^ (n = 2–7) anions were searched and finally optimized at the TPSSh/aug-cc-pVTZ/ECP60MDF level. Besides the smallest cluster of Au_2_Cl_7_^−^, all the most stable isomers in ther series adopt nearly planar zigzag geometries, with both terminal gold atoms in a tetracoordinate Au(III) state and internal gold atoms in Au(I) states. Bond-length analysis shows that only non-terminal Au–Au distances (ca. 2.90–2.92 Å) are much shorter than the van der Waals sum, indicating the existed aurophilic attraction, while terminal distances locted in the range of 3.82–3.90 Å are much longer and show no such interaction. The aurophilic interaction starts at Au_4_Cl_9_^−^ and quickly stabilized for large-size clusters. NBO and ELF analyses confirm that the aurophilic interaction originates from 6s/5d orbital delocalization between adjacent Au(I) centers, with no contribution from Au(III)–Au(I) pairs. Binding energy calculations indicate exothermic growth steps (through the addition of the AuCl unit) with convergence to ca. −175 kJ/mol, and comparison with lower-chloride series shows that the core thermodynamic trend is unaffected by chloride content, though higher chloride substitution accelerates the size convergence. The intensity shift observed for Au_n_Cl_n+m_^−^ (m = 1, 3, 5) suggests that higher laser fluence preferentially stabilizes chlorine-rich species. This is driven by thermodynamically favored growth pathways involving the addition of two Cl atoms. This study shows the structure-directing role of aurophilicity in those hyperchlorinated cluster anions, and provides a comprehensive understanding of the structural evolution and size-dependent aurophilic interactions in high-chloride gold clusters.

## Data Availability

The original contributions presented in the study are included in the article/Supplementary Material, further inquiries can be directed to the corresponding authors.
