# Supplementary material for: Aurophilicity as a structure-directing principle in hyperchlorinated AunCln+5 - (2 ≤ n ≤ 7) clusters
Source: Front Chem. 2026 Oct 1;14:1986508. doi: 10.3389/fchem.2026.1986508 (PMC13669085; doi:10.3389/fchem.2026.1986508)
Supplement: Supplementary file 1 [file Table1.docx]

**SUPPORTING INFORMATION**

Aurophilicity as a Structure-Directing Principle in Hyperchlorinated Au_n_Cl_n+5_^-^ (2 ≤ n ≤ 7) Clusters

Yuqing Chen^1^, Shiyin Xu^1^, Shuo Wang^1^, Shuqi Li^1^, Filip Veljković^2^, Suzana Veličković^2*^, Xianglei Kong^1,3*^

*^1^ State Key Laboratory of Elemento-Organic Chemistry, Frontiers Science Center for New Organic Matter, College of Chemistry, Nankai University, Tianjin 300071, China.*

*^2^ ‘‘VINCA” Institute of Nuclear Sciences - National Institute of the Republic of Serbia, University of Belgrade, Mike Petrovica Alasa 12-14, 11351 Belgrade, Serbia*

*^3^ Tianjin Key Laboratory of Biosensing and Molecular Recognition, College of Chemistry, Nankai University, Tianjin 300071, China*

**Table S1** Natural charges and electron configurations (with the sets of ECP60MDF for Au and aug-cc-pVTZ for Cl) of Au and Cl in Au_n_Cl_n+5_^-^ (n = 2-7).

**Table S2** Calculated electronic energies, zero‑point energies (ZPE), ZPE‑corrected total energies and binding energies of gold‑chlorine clusters.

**Table S1.** Natural charges and electron configurations (with the sets of ECP60MDF for Au and aug-cc-pVTZ for Cl) of Au and Cl in Au_n_Cl_n+5_^-^ (n = 2-7).

| Species | Atom | NPA charge | Electron configuration |
| --- | --- | --- | --- |
| Au_2_Cl_7_^-^-1 | Au2 | 0.47593 | 6S( 0.67)5d( 9.26)6p( 0.56)5f( 0.01) |
|  | Au4 | 0.47335 | 6S( 0.67)5d( 9.26)6p( 0.56)5f( 0.01) |
|  | Cl1 | -0.26880 | 3S( 1.90)3p( 5.35)3d( 0.01) |
|  | Cl3 | -0.09810 | 3S( 1.85)3p( 5.22)3d( 0.01)4p( 0.01) |
|  | Cl5 | -0.33869 | 3S( 1.91)3p( 5.42)3d( 0.01) |
|  | Cl6 | -0.31642 | 3S( 1.90)3p( 5.40)3d( 0.01) |
|  | Cl7 | -0.26848 | 3S( 1.90)3p( 5.35)3d( 0.01) |
|  | Cl8 | -0.32029 | 3S( 1.90)3p( 5.40)3d( 0.01) |
|  | Cl9 | -0.33849 | 3S( 1.91)3p( 5.42)3d( 0.01) |
| Au_3_Cl_8_^-^-1 | Au1 | 0.47082 | 6S( 0.67)5d( 9.27)6p( 0.33)5f( 0.01)7p( 0.23) |
|  | Au4 | 0.37732 | 6S( 0.73)5d( 9.74)6p( 0.13) |
|  | Au5 | 0.47082 | 6S( 0.67)5d( 9.27)6p( 0.33)5f( 0.01)7p( 0.23) |
|  | Cl2 | -0.27096 | 3S( 1.90)3p( 5.35)3d( 0.01) |
|  | Cl3 | -0.23840 | 3S( 1.87)3p( 5.34)3d( 0.01) |
|  | Cl6 | -0.23841 | 3S( 1.87)3p( 5.34)3d( 0.01) |
|  | Cl7 | -0.31885 | 3S( 1.90)3p( 5.40)3d( 0.01) |
|  | Cl8 | -0.33128 | 3S( 1.91)3p( 5.41)3d( 0.01) |
|  | Cl9 | -0.27098 | 3S( 1.90)3p( 5.35)3d( 0.01) |
|  | Cl10 | -0.33124 | 3S( 1.91)3p( 5.41)3d( 0.01) |
|  | Cl11 | -0.31885 | 3S( 1.90)3p( 5.40)3d( 0.01) |
| Au_4_Cl_9_^-^-1 | Au2 | 0.34072 | 6S( 0.73)5d( 9.73)6p( 0.18) |
|  | Au5 | 0.33751 | 6S( 0.73)5d( 9.74)6p( 0.18) |
|  | Au6 | 0.46921 | 6S( 0.68)5d( 9.27)6p( 0.56)5f( 0.01) |
|  | Au9 | 0.46950 | 6S( 0.68)5d( 9.27)6p( 0.56)5f( 0.01) |
|  | Cl1 | -0.22485 | 3S( 1.86)3p( 5.33)3d( 0.01)4p( 0.01) |
|  | Cl3 | -0.34853 | 3S( 1.88)3p( 5.44)3d( 0.02)5p( 0.01) |
|  | Cl4 | -0.26731 | 3S( 1.90)3p( 5.35)3d( 0.01) |
|  | Cl7 | -0.22593 | 3S( 1.86)3p( 5.33)3d( 0.01)4p( 0.01) |
|  | Cl8 | -0.26659 | 3S( 1.90)3p( 5.35)3d( 0.01) |
|  | Cl10 | -0.26659 | 3S( 1.91)3p( 5.40)3d( 0.01) |
|  | Cl11 | -0.31884 | 3S( 1.90)3p( 5.40)3d( 0.01) |
|  | Cl12 | -0.31695 | 3S( 1.90)3p( 5.40)3d( 0.01) |
|  | Cl13 | -0.32422 | 3S( 1.91)3p( 5.40)3d( 0.01) |
| Au_5_Cl_10_^-^-1 | Au2 | 0.29042 | 6S( 0.74)5d( 9.73)6p( 0.23) |
|  | Au5 | 0.34187 | 6S( 0.73)5d( 9.73)6p( 0.18) |
|  | Au6 | 0.46911 | 6S( 0.68)5d( 9.27)6p( 0.56) 5f( 0.01) |
|  | Au9 | 0.46911 | 6S( 0.68)5d( 9.27)6p( 0.56) 5f( 0.01) |
|  | Au15 | 0.34189 | 6S( 0.73)5d( 9.73)6p( 0.18) |
|  | Cl1 | -0.22402 | 3S( 1.86)3p( 5.33)3d( 0.01) 4p( 0.01) |
|  | Cl3 | -0.33211 | 3S( 1.87)3p( 5.43)3d( 0.02) 5p( 0.01) |
|  | Cl4 | -0.26246 | 3S( 1.90)3p( 5.34)3d( 0.01) |
|  | Cl7 | -0.22401 | 3S( 1.86)3p( 5.33)3d( 0.01) 4p( 0.01) |
|  | Cl8 | -0.26246 | 3S( 1.90)3p( 5.34)3d( 0.01) |
|  | Cl10 | -0.32064 | 3S( 1.91)3p( 5.40)3d( 0.01) |
|  | Cl11 | -0.31695 | 3S( 1.90)3p( 5.40)3d( 0.01) |
|  | Cl12 | -0.31698 | 3S( 1.90)3p( 5.40)3d( 0.01) |
|  | Cl13 | -0.32063 | 3S( 1.91)3p( 5.40)3d( 0.01) |
|  | Cl14 | -0.33214 | 3S( 1.87)3p( 5.43)3d( 0.02) 5p( 0.01) |
| Au_6_Cl_11_^-^-1 | Au2 | 0.29594 | 6S( 0.74)5d( 9.73)6p( 0.22) |
|  | Au5 | 0.34064 | 6S( 0.73)5d( 9.73)6p( 0.19) |
|  | Au6 | 0.46935 | 6S( 0.68)5d( 9.27)6p( 0.56)5f( 0.01) |
|  | Au9 | 0.46935 | 6S( 0.68)5d( 9.27)6p( 0.56)5f( 0.01) |
|  | Au14 | 0.29594 | 6S( 0.74)5d( 9.73)6p( 0.22) |
|  | Au15 | 0.34064 | 6S( 0.73)5d( 9.73)6p( 0.19) |
|  | Cl1 | -0.32972 | 3S( 1.87)3p( 5.42)3d( 0.02)5p( 0.01) |
|  | Cl3 | -0.32973 | 3S( 1.87)3p( 5.42)3d( 0.02)5p( 0.01) |
|  | Cl4 | -0.31735 | 3S( 1.91)3p( 5.39)3d( 0.01) |
|  | Cl7 | -0.22358 | 3S( 1.86)3p( 5.33)3d( 0.01)4p( 0.01) |
|  | Cl8 | -0.25899 | 3S( 1.90)3p( 5.34)3d( 0.01) |
|  | Cl10 | -0.25900 | 3S( 1.90)3p( 5.34)3d( 0.01) |
|  | Cl11 | -0.31687 | 3S( 1.90)3p( 5.40)3d( 0.01) |
|  | Cl12 | -0.31735 | 3S( 1.91)3p( 5.39)3d( 0.01) |
|  | Cl13 | -0.31881 | 3S( 1.87)3p( 5.42)3d( 0.02)5p( 0.01) |
|  | Cl16 | -0.22358 | 3S( 1.86)3p( 5.33)3d( 0.01)4p( 0.01) |
|  | Cl17 | -0.31687 | 3S( 1.90)3p( 5.40)3d( 0.01) |
| Au_7_Cl_12_^-^-1 | Au2 | 0.29561 | 6S( 0.74)5d( 9.73)6p( 0.22) |
|  | Au4 | 0.34036 | 6S( 0.72)5d( 9.73)6p( 0.19) |
|  | Au7 | 0.46949 | 6S( 0.68)5d( 9.27)6p( 0.56)5f( 0.01) |
|  | Au12 | 0.30160 | 6S( 0.74)5d( 9.73)6p( 0.22) |
|  | Au13 | 0.29562 | 6S( 0.74)5d( 9.73)6p( 0.22) |
|  | Au15 | 0.34038 | 6S( 0.72)5d( 9.73)6p( 0.19) |
|  | Au17 | 0.46949 | 6S( 0.68)5d( 9.27)6p( 0.56)5f( 0.01) |
|  | Cl1 | -0.31701 | 3S( 1.87)3p( 5.41)3d( 0.02)5p( 0.01) |
|  | Cl3 | -0.32734 | 3S( 1.87)3p( 5.42)3d( 0.02)5p( 0.01) |
|  | Cl5 | -0.22375 | 3S( 1.86)3p( 5.33)3d( 0.01)4p( 0.01) |
|  | Cl6 | -0.25694 | 3S( 1.90)3p( 5.34)3d( 0.01) |
|  | Cl8 | -0.31564 | 3S( 1.91)3p( 5.39)3d( 0.01) |
|  | Cl9 | -0.31556 | 3S( 1.90)3p( 5.40)3d( 0.01) |
|  | Cl10 | -0.31565 | 3S( 1.91)3p( 5.39)3d( 0.01) |
|  | Cl11 | -0.31702 | 3S( 1.87)3p( 5.41)3d( 0.02)5p( 0.01) |
|  | Cl14 | -0.32736 | 3S( 1.87)3p( 5.42)3d( 0.02)5p( 0.01) |
|  | Cl16 | -0.22376 | 3S( 1.86)3p( 5.33)3d( 0.01)4p( 0.01) |
|  | Cl18 | -0.25693 | 3S( 1.90)3p( 5.34)3d( 0.01) |
|  | Cl19 | -0.31559 | 3S( 1.90)3p( 5.40)3d( 0.01) |

**Table S2.** Calculated electronic energies, zero‑point energies (ZPE), ZPE‑corrected total energies and binding energies of gold‑chlorine clusters.

|  | Electronic energy / Hartree | Zero‑point energy (ZPE) / Hartree | ZPE‑corrected total energy / Hartree | Binding energy / Hartree | Binding energy / kJ mol⁻¹ |
| --- | --- | --- | --- | --- | --- |
| AuCl | -595.933635 | 0.000824 | -595.932811 | - | - |
| Au2Cl7- | -3493.344078 | 0.009031 | -3493.335047 | - | - |
| Au3Cl8- | -4089.350194 | 0.010835 | -4089.339359 | -0.071502 | -187.727692 |
| Au4Cl9- | -4685.354428 | 0.012587 | -4685.341841 | -0.069671 | -182.920297 |
| Au5Cl10- | -5281.357590 | 0.014319 | -5281.343271 | -0.068619 | -180.160371 |
| Au6Cl11- | -5877.359380 | 0.016022 | -5877.343358 | -0.067276 | -176.632277 |
| Au7Cl12- | -6473.361415 | 0.018268 | -6473.343147 | -0.066978 | -175.850482 |
| Au2Cl3- | -1652.372175 | 0.003847 | -1652.368328 | - | - |
| Au3Cl4- | -2248.380486 | 0.005467 | -2248.375019 | -0.073880 | -193.972864 |
| Au4Cl5- | -2844.385983 | 0.007371 | -2844.378612 | -0.070782 | -185.837857 |
| Au5Cl6- | -3440.389629 | 0.009135 | -3440.380494 | -0.069071 | -181.346887 |
| Au6Cl7- | -4036.392071 | 0.010910 | -4036.381161 | -0.067856 | -178.154857 |
| Au7Cl8- | -4632.393882 | 0.012687 | -4632.381195 | -0.067224 | -176.495803 |
| Au2Cl5- | -2572.860588 | 0.006476 | -2572.854112 | - | - |
| Au3Cl6- | -3168.866399 | 0.008326 | -3168.858073 | -0.071150 | -186.805433 |
| Au4Cl7- | -3764.870857 | 0.010096 | -3764.860761 | -0.069877 | -183.462253 |
| Au5Cl8- | -4360.873572 | 0.011910 | -4360.861662 | -0.068090 | -178.770484 |
| Au6Cl9- | -4956.875804 | 0.013603 | -4956.862201 | -0.067728 | -177.820132 |
| Au7Cl10- | -5552.877454 | 0.015436 | -5552.862018 | -0.067005 | -175.922578 |
